# Mutational analysis of major IgE-binding epitopes of recombinant bovine αS1-casein

**DOI:** 10.1186/2045-7022-1-S1-P3

**Published:** 2011-08-12

**Authors:** Jean-Charles Gaudin, Hanitra Rabesona, Claudia Nioi, Yvan Choiset, Jean-Marc Chobert, Martine Drouet, Sandra Denery-Papini, Thomas Haertle

**Affiliations:** 1 INRA, BIA, Nantes, France; 2 CHU Angers, Allergologie Generale et Pneumologie, Angers, France

## Background

Cow's milk allergy (CMA) is one of the most widespread human allergies, especially in very young children. One of the main bovine milk allergen is αS1-casein which is considered as intrinsically unfolded protein. Its epitope determinants have been partially characterized using synthetic peptides and crucial amino acid residues for IgE-binding have been identified. However, this very useful approach for epitope characterization neglects local interactions or local structures formed in the full-length protein and their impact on the accessibility of epitopes for IgE.

## Methods

The importance of amino acids previously identified as crucial for IgE-binding was evaluated in the context of the whole protein. The binding of αS1-casein specific IgE from 14 patients with CMA on recombinant αS1-casein and two mutant forms in which critical amino acid residues for IgE-binding have been mutated was measured by indirect ELISA and competitive ELISA.

## Results

For the majority of the patients, mutation reduced significantly IgE-binding but did not suppress it completely. Thus, most of the patients likely produce IgE directed against epitopes, until now, considered as minor. Nevertheless, we observed a great variability in patient responses. For some patients, the complete suppression of IgE-binding was achieved.

## Conclusions

Such recombinant mutated αS1-caseins with reduced IgE-binding ability can be useful for the development of CMA immunotherapy.

